# Use of remote monitoring and integrated platform for the evaluation of sleep quality in adult-onset idiopathic cervical dystonia

**DOI:** 10.1007/s00415-022-11490-4

**Published:** 2022-11-21

**Authors:** Grace A. Bailey, Clare Matthews, Konrad Szewczyk-krolikowski, Peter Moore, Sandra Komarzynski, Elin Haf Davies, Kathryn J. Peall

**Affiliations:** 1 Neuroscience and Mental Health Research Institute, Cardiff University School of Medicine, Hadyn Ellis Building, Maindy Road, Cardiff, CF24 4HQ UK; 2 Aparito Limited, Wrexham, UK; 3 North Bristol NHS Trust, Bristol, UK; 4 The Walton Centre NHS Foundation Trust, Liverpool, UK

**Keywords:** Dystonia, Sleep, Non-motor, Wearable devices, Accelerometry

## Abstract

**Background:**

Up to 70% of individuals diagnosed with adult-onset idiopathic focal cervical dystonia (AOIFCD) report difficulties with sleep. Larger cohort studies using wrist-worn accelerometer devices have emerged as an alternative to smaller polysomnography studies, in order to evaluate sleep architecture.

**Methods:**

To measure activity during the sleep/wake cycle, individuals wore a consumer-grade wrist device (Garmin vivosmart 4) continuously over 7 days on their non-dominant wrist, while completing a daily sleep diary and standardised sleep and non-motor questionnaires via a dedicated app. Sleep measures were derived from the captured raw triaxial acceleration and heart rate values using previously published validated algorithms.

**Results:**

Data were collected from 50 individuals diagnosed with AOIFCD and 47 age- and sex-matched controls. Those with AOIFCD self-reported significantly higher levels of excessive daytime sleepiness (*p* = 0.04) and impaired sleep quality (*p* = 0.03), while accelerometer measurements found the AOIFCD cohort to have significantly longer total sleep times (*p* = 0.004) and time spent in NREM sleep (*p* = 0.009), compared to controls. Overall, there was limited agreement between wearable-derived sleep parameters, and self-reported sleep diary and visual analogue scale records.

**Discussion:**

This study shows the potential feasibility of using consumer-grade wearable devices in estimating sleep measures at scale in dystonia cohorts. Those diagnosed with AOIFCD were observed to have altered sleep architecture, notably longer total sleep time and NREM sleep, compared to controls. These findings suggest that previously reported disruptions to brainstem circuitry and serotonin neurotransmission may contribute to both motor and sleep pathophysiology.

**Supplementary Information:**

The online version contains supplementary material available at 10.1007/s00415-022-11490-4.

## Introduction

Adult-onset idiopathic focal cervical dystonia (AOIFCD), characterised by dystonic posturing localised to the neck, represents the most common form of adult-onset focal dystonia [1]. Associated non-motor symptoms are becoming increasingly recognised, with estimates between 33 to 70% of individuals experiencing sleep impairment with an associated reduction in quality of life [2, 3]. To date, few studies have addressed sleep in AOIFCD using ‘gold-standard’ polysomnography (PSG), with those that have demonstrated reduced sleep efficiency, together with increased sleep latency, wake after sleep onset (WASO), REM sleep latency and percentage of time spent in rapid eye movement (REM) sleep, in comparison to controls [4–6]. However, PSG can be expensive and time consuming, with limitations in being able to extrapolate findings from single night recordings in an unfamiliar environment to the wider context of sleep.

Given the chronic nature of dystonia, greater understanding of sleep in these patients, particularly any potential disruption to normal sleep patterns would require a monitoring system that was low-cost, minimally intrusive and needing minimal input from the patients themselves. In recent years, wrist-worn devices have become widely available, increasingly affordable, and can be used for extended periods of monitoring. The introduction of functions such as heart rate, heart rate variability and raw triaxial acceleration data make such sensors an attractive alternative to PSG. Newer algorithms developed to utilise these features enable differentiation of sleep stages, such as REM and NREM, in addition to more traditional sleep variables [7].

Coupled with accelerometer-based measures, longitudinal symptom understanding can also be aided by Patient-Reported Outcomes (PROs). The use of PROs can improve patient communication with care providers, capturing the patient’s own perceptions and experiences not always addressed in a clinical setting, while also providing a measure of the dynamic symptomatic changes that take place over time [8]. Sleep diaries, the current gold standard for subjective sleep monitoring, are one such example of a PRO, typically completed over a 7-day period [9–11].

Here, we sought to undertake a more detailed investigation of sleep in the largest cohort of individuals diagnosed with AOIFCD to date. Our primary objective was to identify sleep disturbances in individuals diagnosed with AOIFCD by comparing the accelerometer determined sleep variables to those derived from control participants, with secondary measures being to evaluate the concordance between the subjective PROs and objective accelerometer measures within each cohort.

## Methods

### Study cohort

Participants diagnosed with AOIFCD and age- and sex-matched unaffected controls were recruited via the Welsh Movement Disorders Research Network (REC reference: 18/WM/0031). Controls were not bed partners of the patients. Ethical approval for use of consumer-grade wrist-worn wearable and digital PROs completion was obtained from Cardiff University School of Medicine Research Ethics Committee (SOMREC) (Reference: 19/87). Standardised questionnaires were used to collect baseline clinical information, including sex, date of birth, work status, average weekly alcohol use, current medication use and receipt of any ongoing treatment with botulinum toxin injections (BoNT). Participants were required to own an Apple iPhone, the only mobile device able to facilitate the high-volume data capture required when linked with the application (app) outlined below. At least 75 individuals with dystonia were excluded for this reason, as a result Aparito have developed an improved app which facilitates collection of raw triaxial data from Android devices.

### Patient-reported outcomes

**Fig. 1:**
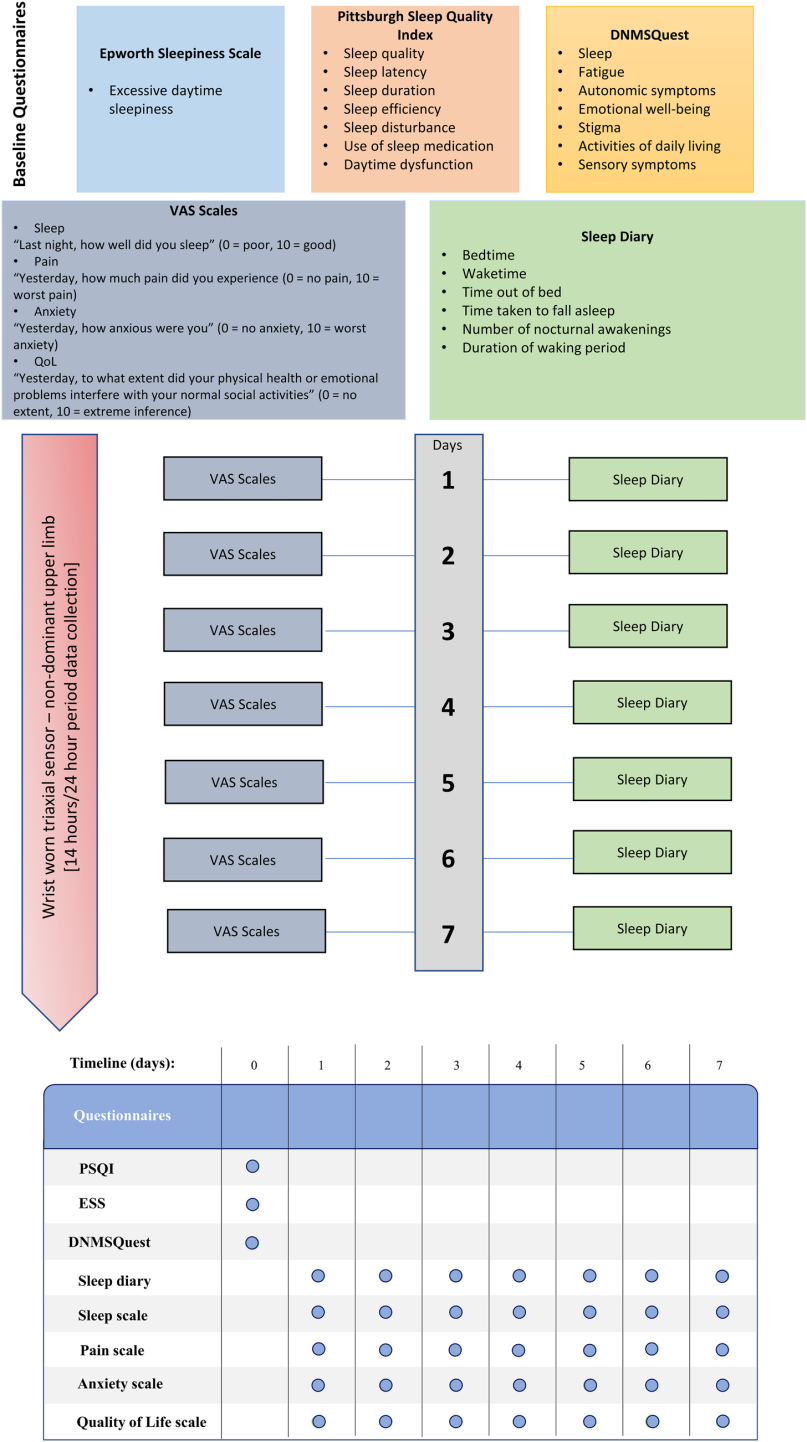
Timeline and frequency of questionnaires. *VAS* visual analogue scale

All PROs were answered via a mobile app (Oxygen by Aparito). At baseline, standardised questionnaires included the Pittsburgh Sleep Quality Index (PSQI) [12], Epworth Sleepiness Scale (ESS) [13] and the dystonia non-motor symptoms questionnaire (DNMSQuest) [14]. Four questions directly relating to dystonia in the DNMSQuest were scored as ‘0’ in the control cohort (Q7, Q10, Q12, Q13). A sleep diary was completed daily for a 7-day period and daily experience of sleep was also assessed using a visual analogue scale (VAS) (0–10). Details and the frequency of the PROs are shown in Fig. 1.

### Wrist-worn device

Participants wore a consumer-grade, microelectromechanical systems (MEMS) triaxial accelerometer and photoplethysmography (PPG) wearable (Vivosmart 4, Garmin) on their non-dominant wrist for 7 days, coinciding with the sleep diary and non-motor symptoms recording. Devices were worn continuously, except for during daily charging. Consumer-grade wearables typically have a minimum of 5 days battery life; however, most wearables only provide processed outputs which have been sampled less frequently (e.g. every minute), this reduces the amount of data transferred and improves battery life. Instead, we sampled raw triaxial data more frequently requiring higher demand which resulted in faster battery usage. Aparito used the Garmin health SDK to access the raw accelerometer data which are not otherwise available via Garmin Connect. A minimum of 14 h/day of recorded data were required for inclusion in onward analysis. Raw triaxial acceleration and heart rate data were collected. To keep focus on sleep and because it was not possible to continuously record over a 24-h period, we did not include L5 and M10 measures of circadian rhythmicity.

### Sleep/wake algorithm

The Walch et al. [15] validated algorithm was used to derive wake/NREM/REM sleep stages from the raw triaxial acceleration and heart rate data. Using these two data parameters, over acceleration alone, logistic regression, *k*-nearest neighbours, a random forest classifier and a neural net were used as models for our comparison (https://github.com/ojwalch/sleep_classifiers) [15] The training of the models was done within either the Walch et al. dataset [15] or the Multi-ethnic Study of Atherosclerosis (MESA) dataset [16, 17]. Walch et al. collected raw triaxial acceleration and heart rate data (via PPG) using the Apple Watch (Series 2 and 3, Apple Inc), a triaxial MEMS accelerometer, and a concurrent night of PSG data (*n* = 31). The MESA dataset consisted of motion data from wrist-worn actigraphy-derived activity counts and a full night of PSG (collected 2010–2012). Actigraphy-derived activity counts were recorded at 1/30 Hz and electrocardiography (ECG) was recorded at 256 Hz. To allow for continuity of outcome parameters, Walch’s raw triaxial data were converted into activity counts using a previously described method [18]. Training of the models has been described elsewhere [15]. The optimal model was then selected for classifying the data collected via the wrist-worn device by the participants diagnosed with AOIFCD and the controls.

### Statistical analysis

Walch’s algorithm was written and run using Python (Python Software Foundation, http://www.python.org). Statistical analyses were performed using R software (version 3.6.3). The overall score of baseline standardised questionnaires (PSQI, ESS, DNMSQuest and Beck’s Depression Inventory (BDI)) were compared using a *t *test or Mann–Whitney *U* statistical approaches. Mean scores of sleep measures from the wrist-worn device and sleep diary were calculated per participant and compared between groups using the Mann–Whitney *U* test [19–23]. Associations between wrist-worn devices and cases/controls were assessed using generalised additive models, and adjusted for work status, weekly alcohol use and use of sleep medication (obtained from PSQI). Agreement between sleep measures derived from the wrist-worn device and sleep diary were assessed using Bland–Altman plots and intraclass correlation coefficient (ICC) and compared using a paired *t *test. We evaluated the relations between the wrist-worn sleep parameters and the sleep PROs using a repeated measures correlation. A *p* value < 0.05 was considered to be significant.

## Results

Fifty individuals diagnosed with AOIFCD (M:14, F: 36, age: 32–73) and 47 age- and sex-matched unaffected controls (M:16, F:31, age: 38–80) (Table 1). Higher rates of prescription of benzodiazepines (*p* = 0.03) and neuropathic agents (*p* = 0.03) were observed in the AOIFCD group compared with controls. Those diagnosed with AOIFCD reported increased levels of excessive daytime sleepiness (*p* = 0.04), as measured by the ESS, and impaired sleep quality (*p* = 0.03) in comparison to controls (Table 2). Further analysis using the PSQI found individuals with AOIFCD to have significantly higher levels of daytime dysfunction (*p* = 0.001) and use of sleep medication (*p* = 0.001). The DNMSQuest revealed overall elevated non-motor symptoms amongst the dystonia cohort (*p* < 0.001), with evidence of increased levels of fatigue (*p* < 0.001), autonomic symptoms (*p* < 0.001), sensory symptoms (*p* < 0.001), stigma (*p* < 0.001), impaired emotional well-being (*p* = 0.001), and impaired activities of daily living (*p* < 0.001). There were no significant differences between AOIFCD and control groups for any of the measures determined using the sleep diary (Table 3).

**Table 1:** Demographics and clinical data of participants

Demographics	AOIFCD	Controls	*p* value
Number of participants	50	47	
Age (median, range)	59 (32 – 73)	61 (38 – 80)	0.13
Sex (%)			
Female	36 (72)	31 (66)	0.67
Male	14 (28)	16 (34)	
Work status (%)			
Employed	23 (46)	17 (36)	0.44
Self-employed	1 (2)	4 (9)	0.32
Volunteering	1 (2)	0 (0)	1
Out of work but not looking	2 (4)	0 (0)	0.5
Retired	21 (42)	24 (51)	0.49
Unable to work	2 (4)	1 (2)	1
Home maker	0 (0)	1 (2)	0.98
Alcohol use (unit/week) (%)			
None	16 (32)	11 (23)	0.47
< 14	23 (46)	27 (57)	0.36
14–21	11 (22)	5 (11)	0.22
> 21	0 (0)	4 (9)	0.11
Medication (%)			
Antidepressants	12 (24)	6 (13)	0.25
Citalopram	2 (4)	1 (2)	
Fluoxetine	1 (2)	2 (4)	
Mirtazapine	1 (2)	1 (2)	
Venlafaxine	2 (4)	0 (0)	
Sertraline	6 (12)	2 (4)	
Benzodiazepines	6 (12)	0 (0)	**0.03**
Clonazepam	4 (8)	0 (0)	
Diazepam	2 (4)	0 (0)	
Neuropathic agents	9 (18)	0 (0)	**0.03**
Amantadine	1 (2)	0 (0)	
Gabapentin	5 (10)	0 (0)	
Nortriptyline	1 (2)	0 (0)	
Pregabalin	2 (4)	0 (0)	
Antihistamine	3 (6)	0 (0)	0.24
Cetirizine	2 (4)	0 (0)	
Promethazine	1 (2)	0 (0)	
Trihexyphenidyl	2 (4)	0 (0)	0.5
Melatonin	1 (2)	0 (0)	1
Zopiclone	0 (0)	1 (2)	0.48
Botulinum toxin (%)	45 (90)	–	

Significant values are reported in bold

**Table 2:** Questionnaire results obtained from AOIFCD and control groups

Questionnaire	AOIFCD	Controls	Effect size (*r*)	*p* value
BDI	*N* = 28	*N* = 22		
BDI score	8 (0–34)	5 (0–31)	0.23	0.17
Number with depression (%)	13 (46)	5 (23)		0.15
PSQI	*N* = 48	*N* = 45		
PSQI score	6.5 (0–15)	5 (1–15)	0.26	**0.03**
Sleep quality score	1 (0–3)	1 (0–3)		0.47
Sleep onset latency score	1 (0–3)	1 (0–3)		0.22
Sleep duration score	0 (0–3)	0 (0–3)		0.76
Sleep efficiency score	1 (0–3)	1 (0–3)		0.88
Sleep disturbance score	1 (0–2)	1 (0–2)		0.07
Use of medication score	0 (0–2)	0 (0–1)		**0.001**
Daytime dysfunction score	1 (0–3)	1 (0–2)		**0.001**
Number with impaired sleep (%)	27 (56)	21 (47)		0.47
ESS	*N* = 49	*N* = 47		
ESS total score	5 (0–17)	4 (0–19)	0.25	**0.04**
Number with abnormal ESS (%)	7 (14)	5 (11)		0.82
Normal (%)	42 (86)	42 (89)		0.82
Mild (%)	3 (6)	3 (6)		1
Moderate (%)	1 (2)	0 (0)		1
Severe (%)	3 (6)	2 (4)		1
DNMSQuest	*N* = 49	*N* = 47		
Total score	7 (0–13)	2 (0–10)	− 0.75	**< 0.001**
Number with impaired sleep (%)	41 (84)	32 (68)		0.08
Impaired sleep quality (%)	33 (67)	27 (57)		0.32
Insomnia (%)	32 (65)	25 (53)		0.23
Number with impaired fatigue (%)	37 (76)	11 (23)		**< 0.001**
Number with autonomic symptoms (%)	24 (49)	6 (13)		< **0.001**
Number with impaired emotional well-being (%)	34 (69)	17 (36)		**0.001**
Number with sensory symptoms (%)	44 (90)	0 (0)		** < 0.001**
Number with impaired AODL (%)	31 (63)	9 (19)		**< 0.001**
Number with stigma (%)	34 (69)	0 (0)		**< 0.001**

Mann–Whitney *U* comparison. Impaired sleep is considered a PSQI global score ≥ 6, abnormal ESS > 11. Depression is considered a BDI score > 10. BDI, PSQI and ESS data are reported as median (range). Significant values are reported in bold

*AOIFCD* adult-onset, isolated focal cervical dystonia, *BDI* Beck’s Depression Inventory, *DNMSQuest* Dystonia Non-Motor Symptom Questionnaire, *ESS* Epworth Sleepiness Scale, *PSQI* Pittsburgh Sleep Quality Index

**Table 3:** Self-reported sleep diary data for AOIFCD and controls

Sleep diary measures	AOIFCD (*n* = 50)	Controls (*n* = 46)	Effect size	*p* value
Sleep onset latency (min)^a^	22.1 (19.8)	14.1 (14.6)	0.22	0.06
Total sleep time (min)^a^	443.9 (68.5)	441.5 (74.6)	− 0.03	0.79
Time in bed (min)^b^	530.4 (70.1)	530 (49.1)	0.001	0.98
Wake after sleep onset (min)^a^	15.1 (29.3)	19.8 (20.5)	− 0.05	0.66
Number of nocturnal awakenings^a^	2 (1.5)	1.9 (0.9)	0.006	0.96
Sleep efficiency (%)^a^	86.5 (13.9)	84.9 (8.2)	0.04	0.79

^a^Mann–Whitney *U* test to compare cases and controls reported as median and interquartile range (IQR) effect size = *r*

^b^*t* test to compare cases and controls reported as mean and standard deviation (SD) effect size = Cohen’s *d*

**Fig. 2:**
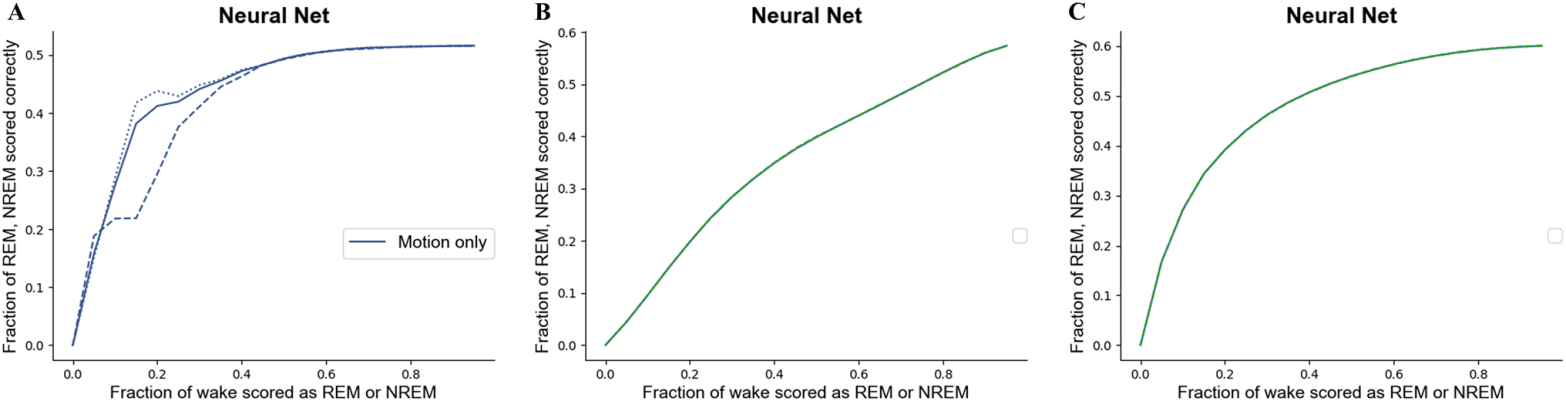
ROC curves in neural network classifiers for classifying wake/NREM/REM using the MESA dataset. **A** Acceleration only, **B** heart rate only**, C** heart rate and acceleration combined**.** The *x*-axis represents the fraction of wake epochs classified incorrectly, with wake epochs classified as either NREM or REM sleep counting as a false positive. The *y-*axis represents REM and NREM accuracy rates. For the heart rate only (**B**) and combined data (**C**) cases, it was possible to choose REM and NREM thresholds to make the accuracies nearly equal, while this was not possible in the acceleration-only feature, instead a binary search was performed to find the value that minimised the differences between REM and NREM accuracy

Using the MESA dataset to train the models to identify wake/NREM/REM sleep stages, the *k*-nearest neighbours, logistic regression and neural net classifiers achieved the same accuracy (0.66) when using combined features (HR and acceleration), however the neural net provided the highest *κ* corresponding value (0.3) and area under curve (AUC) (0.739) values (Supplementary Table 2A). Use of both acceleration and heart rate features also improved (i.e. higher AUC) the classifiers’ ability to differentiate wake/NREM/REM stages (Supplementary Table 2A, Fig. 2), compared to use of a single feature only. The AUC values were higher in classifiers trained with the Walch than MESA dataset (Supplementary Table 2B); however, small sample sizes can reduce the power and accuracy of the model. Based on these data, the neural net classifier trained using acceleration and heart rate data from the MESA dataset were selected to classify our prospectively collected raw triaxial data and heart rate data. The classifier achieved an accuracy of 66% and AUC score of 0.74 when differentiating wake, NREM and REM sleep compared to PSG, similar to previous validated wake/NREM/REM scoring algorithms (accuracy of 73%) [24].

Compared to the control cohort, those with AOIFCD were found to have significantly longer total sleep times (cases: 435 min vs controls: 388 min, *p* = 0.004) and total time spent in NREM (cases: 360 min vs controls: 325 min, *p* = 0.009) compared to controls (Table 4) according to wearable-derived data. When adjusting for average weekly alcohol use, work status and use of sleep medication (PSQI), total sleep time and total NREM sleep remained significant (*p* = 0.039 and *p* = 0.01, respectively). Pearson’s correlation demonstrated no relationship between the time from last BoNT injection and either TST (*r* = − 0.03, *p* = 0.86) or time spent in NREM sleep (*r* = − 0.04, *p* = 0.79).

**Table 4:** Wearable-derived sleep data in AOIFCD patients compared to controls

	AOIFCD (*n* = 48)^a^	Controls (*n* = 43)^a^	Effect size (*r*)^a^	*p* value^a^	*F*^b^	*R*^2b^	*p* value^b^	*F*^c^	*R*^2c^	*p* value^c^
Sleep onset latency (min)	0.96 (1.9)	1 (1.6)	0.05	0.7	1.38	0.03	0.33	1.14	0.06	0.46
Total sleep time (min)	435 (104.4)	388.2 (66.9)	0.35	**0.0038**	3.11	0.09	**0.03**	2.89	0.14	**0.039**
Wake after sleep onset (min)	134.8 (116)	147.4 (51.9)	− 0.15	0.21	1.89	0.01	0.17	1.59	0.05	0.21
Sleep efficiency (%)	75.3 (21.4)	72.6 (9.1)	0.21	0.08	3.31	0.03	0.07	2.99	0.06	0.09
Total REM (min)	61.9 (57.1)	59.2 (41.2)	− 0.05	0.71	0.21	− 0.01	0.65	0.82	0.04	0.37
Total NREM (min)	359.5 (107.4)	325.2 (86.4)	0.32	**0.0089**	6.99	0.06	**0.0097**	6.96	0.11	**0.01**

*CI* confidence interval, *NREM* non-rapid eye movement sleep, *REM* rapid eye movement sleep

^a^Mann–Whitney *U* test to compare cases and controls reported as median and interquartile range (IQR). Significant values are reported in bold

^b^Generalised additive model

^c^Adjusted for work status, alcohol use (unit/week) and use of sleep medication (PSQI measure)

Subjective PROs scores and sensor-derived values differed significantly for all measures with the exception of TST in the dystonia cohort (*p* = 0.81) (Table 5). Both groups overestimated sleep-onset latency (SOL) compared to the wearable device, underestimated WASO, and self-reported higher levels of sleep efficiency, with these values broadly similar between the two groups. Bland–Altman plots for TST, SOL, SE and WASO demonstrate differences between the measures in the dystonia cohort and control groups (Supplementary Fig. 1). Correlation analysis showed no association between wearable-derived sleep parameters and any of the subjectively rated VAS (sleep, pain, anxiety, or quality of life) (*p* > 0.05) (Table 6). Pearson’s correlation demonstrated no relationship between dystonia patients PSQI score and TST (*r* = 0.01, *p* = 0.95) or time spent in NREM sleep (*r* = 0.03, *p* = 0.85), again, ESS scores were not related to TST or NREM (*r* = − 0.22, *p* = 0.14; *r* = − 0.19, *p* = 0.2, respectively).

**Table 5:** Agreement between sleep parameters measured by wearable-device and sleep diary in dystonia cohort

	Wearable-device	Sleep diary	Mean comparison^a^	Effect size^a^	Bland–Altman	Reliability	Correlation^b^
Dystonia cohort				*r*	Limits of agreement	ICC	*r*
Sleep onset latency (min)	0.96 (1.9)	18 (19.9)	**< 0.001**	− 1	− 20.9 to 69.7	0	− 0.002
Total sleep time (min)	435 (104.4)	445 (91.9)	0.81	− 0.004	− 206.8 to 207.9	0.4	0.11
Wake after sleep onset (min)	134.8 (116)	15.7 (30.6)	**< 0.001**	0.95	− 41.8 to 225.2	0.3	0.002
Sleep efficiency (%)	75.3 (21.4)	86 (12.4)	**< 0.001**	− 1	61.4 to 102.6	0	0.09
Control cohort							
Sleep onset latency (min)	1 (1.6)	15 (18.9)	**< 0.001**	− 1	− 44.5 to 97.8	0	− 0.04
Total sleep time (min)	388.2 (66.9)	443.5 (82.3)	**0.005**	− 0.49	− 219.1 to 275.7	0.1	0.04
Wake after sleep onset (min)	147.4 (51.9)	20 (23.2)	**< 0.001**	0.94	− 248 to 34.7	0	0.2
Sleep efficiency (%)	72.6 (9.1)	84 (7.5)	**< 0.001**	− 1	60.6 to 102.2	0	0.08

*p*-values in bold indicate those that are statistically significant

*ICC* intraclass correlation coefficient

^a^Wilcoxon signed rank test was used for mean comparison of non-normally distributed data, reported as median and interquartile range (IQR)

^b^Repeated measures correlation

**Table 6:** Repeated measures correlation coefficient between wearable-derived and subjective VAS

Wearable-device parameters	AOIFCD (*n* = 45)		Control (*n* = 43)	
	Sleep VAS (*r*)	*p* value	Sleep VAS (*r*)	*p* value
Sleep onset latency	0.13	0.11	0.06	0.53
Total sleep time	− 0.07	0.38	0.008	0.93
Wake after sleep onset	− 0.03	0.69	0.004	0.97
Sleep efficiency	0.01	0.99	0.05	0.59
NREM (min)	− 0.07	0.35	0.11	0.26
REM (min)	0.05	0.53	0.17	0.06

	Anxiety VAS (*r*)	*p* value	Anxiety VAS (*r*)	*p* value
Sleep onset latency	0.01	0.86	0.01	0.89
Total sleep time	0.05	0.49	0.001	0.99
Wake after sleep onset	− 0.11	0.17	− 0.03	0.69
Sleep efficiency	0.09	0.23	0.05	0.53
NREM (min)	0.05	0.56	0.07	0.38
REM (min)	0.03	0.56	− 0.02	0.82

	Pain VAS (*r*)	*p* value	Pain VAS (*r*)	*p* value
Sleep onset latency	0.04	0.59	− 0.02	0.84
Total sleep time	0.03	0.7	− 0.05	0.56
Wake after sleep onset	− 0.01	0.93	− 0.08	0.35
Sleep efficiency	0.01	0.94	0.07	0.41
NREM (min)	− 0.02	0.83	− 0.04	0.63
REM (min)	− 0.02	0.83	0.08	0.36

	QoL VAS (*r*) (*n* = 44)	*p* value	QoL VAS (*r*) (*n* = 42)	*p* value
Sleep onset latency	− 0.1	0.26	− 0.03	0.76
Total sleep time	− 0.02	0.8	− 0.01	0.91
Wake after sleep onset	− 0.02	0.86	− 0.03	0.77
Sleep efficiency	− 0.01	0.87	0.02	0.82
NREM (min)	− 0.01	0.9	− 0.07	0.47
REM (min)	− 0.02	0.78	0.08	0.36

*NREM* non-rapid eye movement sleep, *REM* rapid eye movement, *QoL* quality of life, *VAS* visual analogue scale

## Discussion

These findings represent the largest study of wrist-worn consumer-grade devices (accelerometer and PPG) in a cohort of individuals diagnosed with AOIFCD to date. We evaluated objective and subjective sleep measures using a consumer-grade wrist-worn wearable device, validated sleep questionnaires (PSQI, ESS, DNMSQuest) and sleep diary in both the recruited AOIFCD cohort and matched control group. Wearable-device derived sleep measurements found those diagnosed with AOIFCD experienced significantly longer TST and increased time spent in NREM sleep compared to controls, with these differences replicated in the questionnaire captured data with higher PSQI and ESS scores in dystonia compared to control groups. As anticipated, the DNMSQuest scores were significantly higher in the dystonia cohort compared to controls, with this also including higher levels of fatigue. There was poor agreement between total sleep time derived from the wearable-device and the sleep diary in the dystonia cohort not mirrored in the control group. No other sleep parameters obtained from the wearable-device were comparable to the sleep diary, nor correlated with any of the subjectively rated VAS.

Data collected using the DNMSQuest questionnaire confirmed a significantly higher rate of non-motor symptoms in the AOIFCD group compared to controls. Of those with AOIFCD, 98% (48/49) individuals reported at least one NMS and 76% (37/49) described five or more symptom types. Fatigue was the most prominent sleep-related impairment (76%); however, there was also evidence of significantly higher rates of sensory symptoms (90 and 0%, *p* < 0.001) and impaired emotional well-being (69 and 36%, *p* = 0.001) (Table 2). Interestingly, scores relating to sleep quality and insomnia were not reported to be significantly different between the groups (cases: 67% and controls: 57%, *p* = 0.32, cases: 65 vs 53%, *p* = 0.23). These findings may in part be accounted for in the design of the DNMSQuest, a questionnaire aimed at determining the burden of NMS in dystonia cohorts, rather than for use in the specific identification of sleep disturbance or sleep disorders. Interestingly, we found evidence of excessive daytime sleepiness as measured by the ESS and impaired sleep quality assessed by using the PSQI in the AOIFCD cohort compared to controls. Prior studies have also found inconsistencies in self-reported sleep symptoms by those diagnosed with dystonia, with a single study reporting excessive daytime sleepiness in those with cervical dystonia measured using the ESS [25], while the majority of other studies have identified no excess sleepiness [26–29]. Use of the PSQI questionnaire has again found conflicting results with some reporting higher overall scores in dystonia cohorts compared to controls [26, 27, 29], whereas others have found comparable scores to controls [30], particularly when controlling for symptoms of depression and anxiety [31].

Total sleep time and time spent in NREM sleep derived from the wearable-device recordings were significantly increased amongst the AOIFCD cohort compared to controls, with these differences still observed when adjusting for alcohol use (units/week), work status and use of sleep medication. BDI scores were not included in this model due to the low completion level; however, further assessments of mood would in key in any future studies. Of the few polysomnographic studies to date, PSG-studies have identified no differences in TST between cervical dystonia and control cohorts [4, 5, 32], although evidence suggests reduced muscle activity when in a relaxed state (i.e. lying down) in those with cranio-cervical dystonia which may be misclassified as a sleeping state [4]. Of these two studies, a total sleep time closely reflecting our results (470.5 min) was identified amongst a younger cohort (mean age: 42.8 years) [5], while in comparison to findings shown here reduced TST (368.6 min) were reported in a cohort closer matched in age (50.5 years) [4]. A recent study investigating the pathophysiological effects of BoNT treatment on sleep have also shown TST estimated using actigraphy was comparable to controls, although small sample size may explain observed differences (*n* = 6) [32]. In line with our findings of increased NREM sleep in the AOIFCD cohort, a single study noted increased N1 sleep in those with cranial dystonia [33], with a second trending towards an increased N1 percentage (*p* = 0.079) [5]. The serotonergic raphe and brainstem are typically involved in NREM maintenance, both of which have been implicated in dystonia pathophysiology. Interestingly, imaging studies have also demonstrated an association between increased sleep disturbance and higher serotonergic binding potential in the dorsal raphe nucleus, caudate nucleus and hippocampus [34]. These findings suggest that shared underlying mechanisms may give rise to the abnormal sleep architecture observed in this study and the motor features evident in AOIFCD.

Interestingly, PSG-based studies have previously reported REM sleep change, including increased REM sleep latency and reduced REM sleep percentage in patients with cervical dystonia [4, 5]; however, several oral medical therapies used in the management of the motor symptoms of dystonia (e.g. trihexyphenidyl and benzodiazepines) have been linked with decreased REM sleep duration [35, 36], with these studies reporting 48% of the cohort prescribed trihexyphenidyl and 24% prescribed clonazepam [5]. By contrast, only 8 and 4% of the cohort recruited in this study were prescribed clonazepam or trihexyphenidyl, respectively, likely due to the majority being recruited via neurotoxin services, with their motor symptoms predominantly managed through injectable botulinum toxin with this potentially explaining the lack of observable difference in REM sleep duration.

Significant differences were noted between wearable and diary estimates of the sleep variables across both cohorts, whereby the sleep diaries overestimated SOL and underestimated WASO when compared to the wearable-device, consistent with previous sleep studies of other neuropsychiatric disorders [37]. Methodological differences potentially contribute to a portion of these discrepancies, for example subjective recall may not be as accurate in ageing adults and those with cognitive impairment, with previous studies of AOIFCD indicating impairments to executive function [38]. In addition, temporal differences are also likely to contribute, with individual recall of nocturnal awakenings differing substantially from mathematical evaluation of 30-s epochs [39].

In addition to standardised questionnaires, we used daily VAS to examine perceptions of sleep quality. A single-item sleep quality assessment is practical when measures are taken frequently. Here, the sleep VAS did not correlate with any wearable measures which suggests that a single Likert scale rating sleep is not sufficiently detailed to capture more subtle sleep abnormalities. Despite this, several studies have validated single-item measures of sleep quality [40]. Sleep quality is a complex phenomenon involving multiple aspects making measurement difficult, especially given that how individuals determined sleep quality was not specifically addressed here. A previous study in older adults also highlighted that perceived sleep quality differs from that of objective sleep quality [41], potentially indicating the need to not only consider both collectively, but also independently.

One of the main limitations of this study was the lack of simultaneously captured PSG data, the gold standard method for evaluating sleep. As a result, we were unable to compare the sensitivity and specificity of the sleep model used in our analysis. Future studies of dystonia cohorts should aim to include weeklong sleep diary and actigraphy measurements, together with an overlapping night of PSG measurements to allow for epoch-by-epoch validation. Other elements to consider with this study include that the wearable device used was consumer grade and may not be as accurate as validated actigraphs; however, rather than use brand-specific algorithms that are not publicly available, we were able to access the raw triaxial acceleration data for direct data analysis, and incorporated heart rate data into the analysis over acceleration alone, improving the generalisability of our results with other studies. Finally, we did not control for the time period between BoNT injections and data capture, with neurotoxin injections potentially directly impacting sleep quality as well as indirectly through improved pain management. However, it should be noted that work to date suggests a non-concordant relationship between sleep and motor symptoms, with no improvement to sleep quality with improved motor symptom management [28].

This study demonstrates the feasibility of wearable devices in estimating sleep measures, at scale, amongst those diagnosed with AOIFCD, identifying both reduced sleep quality and altered sleep architecture, namely increased duration of TST and NREM sleep. We have also shown the benefits of using data from mobile devices in enabling the measurement of sleep stages at scale, and their objective value when compared to subjective participant reported sleep quality using standardised sleep questionnaires, PROs, sleep diaries and VAS scoring. Our work and previous PSG studies indicate a need for further evaluation of sleep across the many types of dystonia, as well as the incorporation of sleep assessment into routine clinical care.

## Supplementary Information

Below is the link to the electronic supplementary material.

Supplementary file1Supplementary Fig. 1: Bland–Altman plots for (A) sleep onset latency, (B) total sleep time, (C) wake after sleep onset and (D) sleep efficiency in i) dystonia cohort and ii) control cohort. The y-axis represents the difference between the two measures (diary—wearable) and the x-axis shows the average of both measures. The central dashed horizontal line represents the mean difference between both methods, accompanied by two horizontal dashed lines that demonstrate the 95% limits of agreement (mean difference ± 1.96 standard deviation). SE: Sleep efficiency, SOL: Sleep onset latency, TST: Total sleep time, WASO: Wake after sleep onset (TIF 2916 KB)

Supplementary file2 (DOCX 16 KB)

Supplementary file3 (DOCX 18 KB)

## Data Availability

The data that support the findings of this study are available on request from the corresponding author.

## Data access statement

Data underpinning the results presented here are available on request.
