# Supplementary figures and images for: Use of remote monitoring and integrated platform for the evaluation of sleep quality in adult-onset idiopathic cervical dystonia
Source: J Neurol. 2022 Nov 21;270(3):1759–69. doi: 10.1007/s00415-022-11490-4 (PMC9971061; doi:10.1007/s00415-022-11490-4)

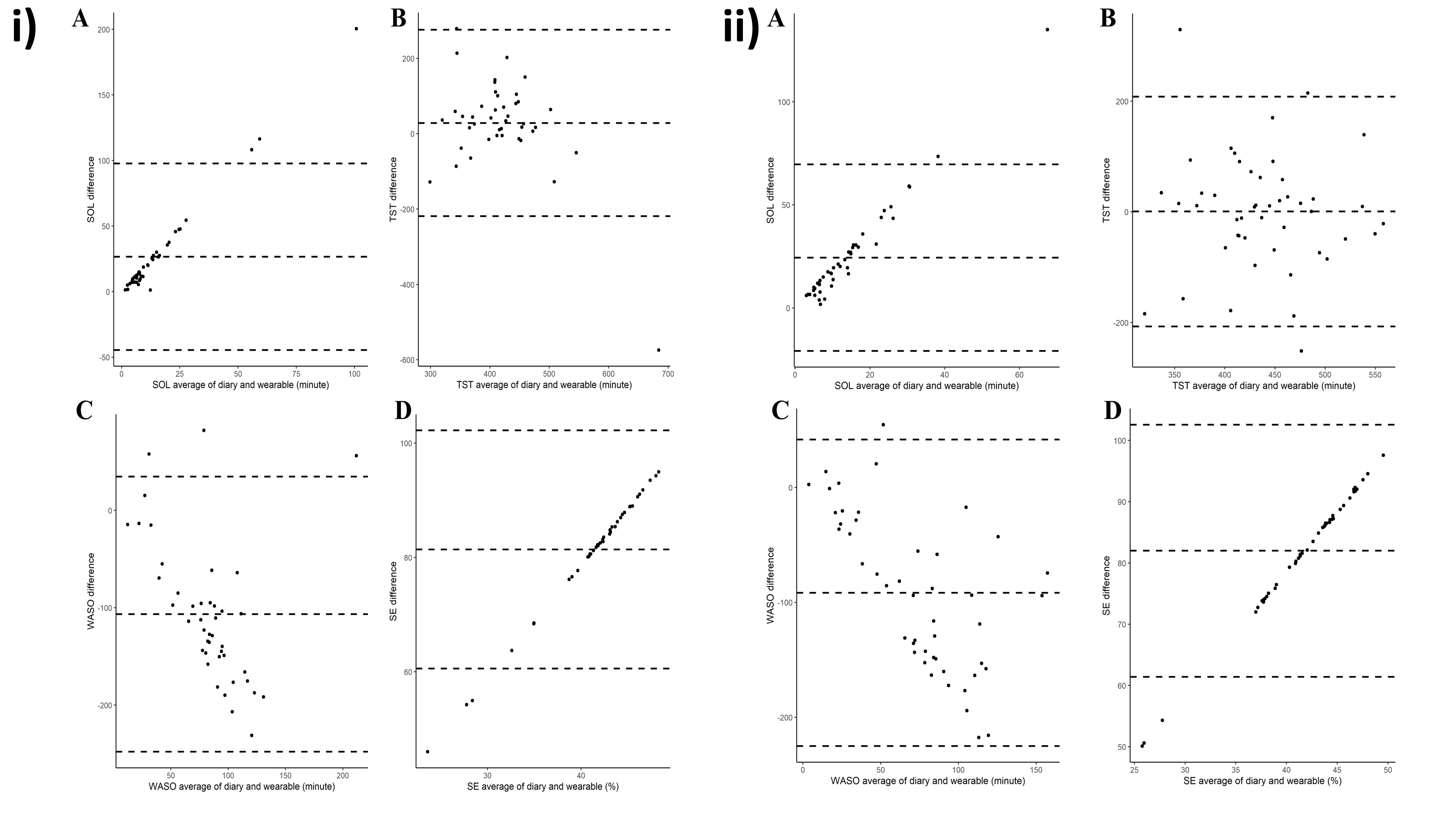

Supplement: Supplementary file 1 — Supplementary file1Supplementary Fig. 1: Bland–Altman plots for (A) sleep onset latency, (B) total sleep time, (C) wake after sleep onset and (D) sleep efficiency in i) dystonia cohort and ii) control cohort. The y-axis represents the difference between the two measures (diary—wearable) and the x-axis shows the average of both measures. The central dashed horizontal line represents the mean difference between both methods, accompanied by two horizontal dashed lines that demonstrate the 95% limits of agreement (mean difference ± 1.96 standard deviation). SE: Sleep efficiency, SOL: Sleep onset latency, TST: Total sleep time, WASO: Wake after sleep onset (TIF 2916 KB) [file 415_2022_11490_MOESM1_ESM.tif]
