# Supplementary material for: Use of remote monitoring and integrated platform for the evaluation of sleep quality in adult-onset idiopathic cervical dystonia
Source: J Neurol. 2022 Nov 21;270(3):1759–69. doi: 10.1007/s00415-022-11490-4 (PMC9971061; doi:10.1007/s00415-022-11490-4)
Supplement: Supplementary file 2 — Supplementary file2 (DOCX 16 KB) [file 415_2022_11490_MOESM2_ESM.docx]

**Supplementary Table 1.** Breakdown of medications used by both cohorts

| **Medications n(%)** |  | | **AOIFCD (n=50)** | | **Controls (n=47)** | | | |
| --- | --- | --- | --- | --- | --- | --- | --- | --- |
| Amantadine |  |  | | 1 (2) |  |  | 0 (0) |  |
| Amitriptyline |  |  | | 4 (8) |  |  | 1 (2) |  |
| Atenolol |  |  | | 1 (2) |  |  | 0 (0) |  |
| Bisoprolol |  |  | | 0 (0) |  |  | 1 (2) |  |
| Cetirizine |  |  | | 2 (4) |  |  | 0 (0) |  |
| Circadin (melatonin) |  |  | | 1 (2) |  |  | 0 (0) |  |
| Citalopram |  |  | | 2 (4) |  |  | 1 (2) |  |
| Clonazepam |  |  | | 4 (8) |  |  | 0 (0) |  |
| Diazepam |  |  | | 2 (4) |  |  | 0 (0) |  |
| Fluoxetine |  |  | | 1 (2) |  |  | 2 (4) |  |
| Gabapentin |  |  | | 5 (10) |  |  | 0 (0) |  |
| Hyoscine |  |  | | 1 (2) |  |  | 0 (0) |  |
| Mirtazapine |  |  | | 1 (2) |  |  | 1 (2) |  |
| Nortriptyline |  |  | | 1 (2) |  |  | 0 (0) |  |
| Pregabalin |  |  | | 2 (4) |  |  | 0 (0) |  |
| Promethazine |  |  | | 1 (2) |  |  | 0 (0) |  |
| Propranolol |  |  | | 2 (4) |  |  | 0 (0) |  |
| Sertraline |  |  | | 6 (12) |  |  | 2 (4) |  |
| Topiramate |  |  | | 0 (0) |  |  | 1 (2) |  |
| Trihexyphenidyl |  |  | | 2 (4) |  |  | 0 (0) |  |
| Venlafaxine |  |  | | 2 (4) |  |  | 0 (0) |  |
| Zopiclone |  |  | | 0 (0) |  |  | 1 (2) |  |
| Botulinum toxin |  |  | | 45 (90) |  |  | - |  |
