# Supplementary material for: Use of remote monitoring and integrated platform for the evaluation of sleep quality in adult-onset idiopathic cervical dystonia
Source: J Neurol. 2022 Nov 21;270(3):1759–69. doi: 10.1007/s00415-022-11490-4 (PMC9971061; doi:10.1007/s00415-022-11490-4)
Supplement: Supplementary file 3 — Supplementary file3 (DOCX 18 KB) [file 415_2022_11490_MOESM3_ESM.docx]

**Supplementary Table 2A.** Sleep stage classification accuracy across different classifiers using the MESA dataset

| **Classifier** | **Features** | **Accuracy** | **Wake correct** | **NREM correct** | **REM correct** | **κ** | **AUC** |
| --- | --- | --- | --- | --- | --- | --- | --- |
| Logistic regression | HR | 0.605 | 0.6 | 0.352 | 0.353 | 0.125 | 0.611 |
|  | Acceleration | 0.691 | 0.6 | 0.572 | 0.36 | 0.436 | 0.738 |
|  | HR and acceleration | 0.658 | 0.6 | 0.474 | 0.473 | 0.282 | 0.716 |
| *k*-nearest neighbours | HR | 0.577 | 0.6 | 0.299 | 0.299 | 0.045 | 0.584 |
|  | Acceleration | 0.677 | 0.6 | 0.445 | 0.49 | 0.416 | 0.705 |
|  | HR and acceleration | 0.655 | 0.6 | 0.496 | 0.495 | 0.29 | 0.725 |
| Random forest | HR | 0.603 | 0.6 | 0.351 | 0.352 | 0.124 | 0.620 |
|  | Acceleration | 0.691 | 0.6 | 0.572 | 0.36 | 0.437 | 0.739 |
|  | HR and acceleration | 0.602 | 0.6 | 0.497 | 0.495 | 0.274 | 0.733 |
| Neural net | HR | 0.603 | 0.6 | 0.349 | 0.350 | 0.128 | 0.619 |
|  | Acceleration | 0.691 | 0.6 | 0.475 | 0.463 | 0.436 | 0.738 |
|  | HR and acceleration | 0.662 | 0.6 | 0.507 | 0.507 | 0.299 | 0.739 |

**Abbreviation**: AUC: Area under curve, HR: Heart rate, κ: Cohen’s kappa coefficient of agreement

NREM and REM correct relate to the fraction of NREM and REM epochs scored correctly when a threshold is chosen so they are as close as possible, while maintaining the fraction of correctly scored wake epochs at 0.6

**Supplementary Table 2B.** Sleep stage classification accuracy across different classifiers using the Walch dataset

| **Classifier** | **Features** | **Accuracy** | **Wake correct** | **NREM correct** | **REM correct** | **κ** | **AUC** |
| --- | --- | --- | --- | --- | --- | --- | --- |
| Logistic regression | HR | 0.691 | 0.6 | 0.390 | 0.390 | 0.035 | 0.655 |
|  | Acceleration | 0.706 | 0.6 | 0.542 | 0.294 | 0.090 | 0.724 |
|  | HR and acceleration | 0.702 | 0.6 | 0.578 | 0.578 | 0.096 | 0.747 |
| *k*-nearest neighbours | HR | 0.668 | 0.6 | 0.365 | 0.364 | 0.070 | 0.641 |
|  | Acceleration | 0.691 | 0.6 | 0.582 | 0.278 | 0.068 | 0.655 |
|  | HR and acceleration | 0.696 | 0.6 | 0.565 | 0.566 | 0.141 | 0.753 |
| Random forest | HR | 0.673 | 0.6 | 0.376 | 0.376 | 0.216 | 0.659 |
|  | Acceleration | 0.694 | 0.6 | 0.520 | 0.332 | 0.084 | 0.709 |
|  | HR and acceleration | 0.660 | 0.6 | 0.579 | 0.579 | 0.242 | 0.775 |
| Neural net | HR | 0.701 | 0.6 | 0.391 | 0.391 | 0.031 | 0.678 |
|  | Acceleration | 0.707 | 0.6 | 0.348 | 0.562 | 0.090 | 0.700 |
|  | HR and acceleration | 0.712 | 0.6 | 0.592 | 0.593 | 0.112 | 0.774 |

**Abbreviation**: AUC: Area under curve, HR: Heart rate, κ: Cohen’s kappa coefficient of agreement

NREM and REM correct relate to the fraction of NREM and REM epochs scored correctly when a threshold is chosen so they are as close as possible, while maintaining the fraction of correctly scored wake epochs at 0.6
